# Fish Oil Improves Pathway-Oriented Profiling of Lipid Mediators for Maintaining Metabolic Homeostasis in Adipose Tissue of Prediabetic Rats

**DOI:** 10.3389/fimmu.2021.608875

**Published:** 2021-04-21

**Authors:** Gabriel Dasilva, Salomé Lois, Lucía Méndez, Bernat Miralles-Pérez, Marta Romeu, Sara Ramos-Romero, Josep L. Torres, Isabel Medina

**Affiliations:** ^1^Food Science Department, Instituto de Investigaciones Marinas (IIM-CSIC), Vigo, Spain; ^2^Unitat de Farmacologia, Facultat de Medicina i Ciències de la Salut, Universitat Rovira i Virgili, Reus, Spain; ^3^Biological Chemistry Department, Instituto de Química Avanzada de Catalunya (IQAC-CSIC), Barcelona, Spain

**Keywords:** adipose tissue, prediabetes, inflammation, ω3 lipid mediators, specialized resolvers

## Abstract

Adipose tissue is now recognized as an active organ with an important homeostatic function in glucose and lipid metabolism and the development of insulin resistance. The present research investigates the role of lipid mediators and lipid profiling for controlling inflammation and the metabolic normal function of white adipose tissue from rats suffering from diet-induced prediabetes. Additionally, the contribution to the adipose lipidome induced by the consumption of marine ω-3 PUFAs as potential regulators of inflammation is addressed. For that, the effects on the inflammatory response triggered by high-fat high-sucrose (HFHS) diets were studied in male Sprague-Dawley rats. Using SPE-LC-MS/MS-based metabolo-lipidomics, a range of eicosanoids, docosanoids and specialized pro-resolving mediators (SPMs) were measured in white adipose tissue. The inflammatory response occurring in prediabetic adipose tissue was associated with the decomposition of ARA epoxides to ARA-dihydroxides, the reduction of oxo-derivatives and the formation of prostaglandins (PGs). In an attempt to control the inflammatory response initiated, LOX and non-enzymatic oxidation shifted toward the production of the less pro-inflammatory EPA and DHA metabolites rather than the high pro-inflammatory ARA hydroxides. Additionally, the change in LOX activity induced the production of intermediate hydroxides precursors of SPMs as protectins (PDs), resolvins (Rvs) and maresins (MaRs). This compensatory mechanism to achieve the restoration of tissue homeostasis was significantly strengthened through supplementation with fish oils. Increasing proportions of ω-3 PUFAs in adipose tissue significantly stimulated the formation of DHA-epoxides by cytochrome P450, the production of non-enzymatic EPA-metabolites and prompted the activity of 12LOX. Finally, protectin PDX was significantly reduced in the adipose tissue of prediabetic rats and highly enhanced through ω-3 PUFAs supplementation. Taken together, these actively coordinated modifications constitute key mechanisms to restore adipose tissue homeostasis with an important role of lipid mediators. This compensatory mechanism is reinforced through the supplementation of the diet with fish oils with high and balanced contents of EPA and DHA. The study highlights new insides on the targets for effective treatment of incipient diet-induced diabetes and the mechanism underlying the potential anti-inflammatory action of marine lipids.

## Introduction

Adipose tissue is not considered anymore as an inert reservoir for energy but an immune organ with endocrine, paracrine and autocrine functions (1). Adipocytes regulate fat mass and nutrient homeostasis. They are also implicated in hemostasis, blood pressure control, the immune response, bone mass, and thyroid and reproductive function (2). In the context of obesity, white adipose tissue plays an important homeostatic role in glucose and lipid metabolism; and the development of insulin resistance is now recognized to be initiated by inflammation of the adipose tissue (3). The initial events giving rise to this adipose tissue inflammation are not well-known yet, because of the complex combination of endocrine and immune factors that actively act to modulate this microenvironment. Adipocyte dysfunctions are now considered essential to explain the low-chronic level of systemic inflammation linked to diet-induced metabolic diseases (4). In metabolically unhealthy obesity, the storage capacity of adipocytes is exceeded, and further caloric overload leads to fat accumulation in ectopic tissues and visceral adipose depots, an event commonly defined as “lipotoxicity” (5). It has been largely demonstrated that excessive lipid accumulation in ectopic tissues leads to local inflammation and insulin resistance. Additionally, when adipocytes reach a cell and tissue expansion limitation, an inflammatory program in response to this stress is initiated (4). Indeed, a high-energy state triggers uncontrolled inflammatory responses in white adipose tissue, leading to chronic low-grade inflammation and therefore fostering the progression of insulin resistance. Within this context, a large amount of evidence indicates that the presence of persistent un-resolved adipose inflammation is addressed by a deregulated balance between the synthesis and release of pro-inflammatory lipid and peptide mediators by adipocytes and reduced levels of anti-inflammatory molecules (6, 7).

Bioactive lipid mediators have been increasingly recognized as important endogenous regulators of key cellular processes triggering inflammation (8). ω-6 polyunsaturated fatty acids (PUFAs) and especially arachidonic acid (ARA), are the prime precursors for the biosynthesis of inflammatory lipid mediators. Except for lipoxins, the majority of ARA derived eicosanoids have pro-inflammatory properties. On the other hand, bioactive lipid mediators generate from the ω-3 PUFAs, eicosapentaenoic acid (EPA) and docosahexaenoic acid (DHA) from fish oils, are capable of decreasing inflammation and the recruitment of polymorphonuclear leukocyte in many inflammatory disease models (8). According to the latest research, the programming of inflammation resolution can be governed by a specialized class of lipid mediators derived from the metabolism of long-chain PUFAs (8, 9). Specialized pro-resolving lipid mediators (SPMs) – generated from DHA and EPA as protectins (PDs), resolvins (Rvs) and maresins (MaRs) – can help stop the cycle leading to unremitting inflammation, protect organs, and stimulate tissue regeneration (10). They coordinately act to regulate epithelial, endothelial, and immune cell function for the restoration of homeostasis in a specific time-limited manner (11). Notably, a deficit in the biosynthesis of SPMs has recently been uncovered in inflamed obese adipose tissue in which resolvins, RvD1 and RvD2, were able to rescue impaired expression and secretion of adiponectin as well as decreasing pro-inflammatory adipokine production (12). These SPMs have been shown to improve insulin sensitivity in obese diabetic mice and attenuate age-associated adiposity (13).

Strategies for anti-inflammatory nutrition have been largely focused on decreasing the content of dietary ω-6 PUFAs, saturated fats, and refined carbohydrates that may induce inflammatory responses, and increasing ω-3 PUFAs and antioxidants that activate endogenous mechanisms to reduce inflammation (14). Recently, several pieces of information are starting to emerge in supporting the increased consumption of EPA and DHA to exert a beneficial effect on white adipose tissue function and metabolism (15). Previous studies demonstrated that fish oil supplements given to obese human subjects decreased expression of inflammasome-associated IL-18 and IL-1β and circulating IL-18 levels, and other adipose inflammatory genes (16). Saitoh et al. (17) have reported that supplementation with fish oil reduced macrophage infiltration and cytokine levels in mice white adipose tissue contributing to inhibit the insulin resistance caused by a high-fat diet through an anti-inflammatory effect. However, other studies have shown that chronic supplementation of fish oil might alter lipid signaling in obese adipose tissue suggesting dysregulation of adipose tissue expansion and inflammatory signaling (18). Lack of self-resolution, as well as dysregulation in the utilization of supplementary EPA and DHA for the synthesis of anti-inflammatory lipid mediators, have been also pointed out (18). The need for current studies supporting the mechanistic role of EPA and DHA as potent modulators of adipose tissue and adipocyte function has been lately indicated (15). Previous works have demonstrated that supplementation with fish oil resulted in a lower concentration of plasma ARA pro-inflammatory lipid mediators in rats fed high-fat high-sucrose diets (HFHS) and favored the activity of specific antioxidant enzymes related to oxidative stress as glutathione peroxidase (GPx) (19).

The present research is aimed to investigate the role of ω-3 and ω-6 PUFAs and the participation of bioactive ω-3-derived lipid mediators in the resolution of adipose tissue inflammation and the maintenance of metabolic normal function in prediabetic Sprague-Dawley rats. The pathway oriented profiling of pro-inflammatory and pro-atherogenic eicosanoids and docososanoids together with the formation of SPMs is discussed to illustrate their effect to maintain the adipose tissue homeostasis during overnutrition. Then, the contribution of marine ω-3 PUFAs supplementation to shift adipose lipidome and ameliorate the low grade of inflammation of prediabetic adipose tissue is considered. Male Sprague-Dawley rats fed HFHS diets were compared with controls fed a standard diet (STD). Fish oil in a balanced 1:1 EPA/DHA proportion was supplemented in both dietary frameworks. A lipidomic platform based on SPE-LC-MS/MS was applied to determine the influence of hyperenergetic diets and fish oil supplementation on the synthesis of ω-3 and ω-6 eicosanoids and docosanoids. The pathway oriented profiling of lipid mediators, the incorporation of ω-3 PUFAs into the adipose tissue fatty acids and the fatty acid desaturases were discussed. Finally, ectopic lipid deposition so as plasma markers of inflammation and lipid and carbohydrate metabolism were associated with a shift in the adipose tissue lipidome resulting in dysregulation of eicosanoid and docosanoid mediators.

## Material and Methods

### Animals and Diets

Thirty-six male 8-9 weeks-old Sprague-Dawley rats weighing about 322 ± 18 g (Harlan Laboratories Ltd., UK.) were kept in an insulated room with a constantly regulated temperature (22 ± 2°C) and controlled humidity (50 ± 10%) in a 12 h artificial light cycle. The rats were randomized into four groups (9 rats per group), and fed for 21 weeks one of the following diets: (a) a standard diet (2014 Teklad Global 14% Protein Diet from Envigo, IN, USA, STD control group), (b) a STD diet supplemented with fish oil EPA/DHA in a balanced 1:1 ratio (STD+ω3) (c) a HFHS diet (TD.08811 45% kcal fat diet from Envigo, IN, USA, HFHS control group), and (d) a HFHS diet supplemented with fish oil EPA/DHA in a balanced 1:1 ratio (HFHS+ω3). Rats had *ad libitum* access to water (Ribes, Barcelona, Spain) and food. Both daily water and food intakes were recorded throughout the experimental intervention. The experimental diets were described in the supplementary material and are the same used for their research by Muñoz et al. (20) (**Supplementary Table S1**).

Adequate amounts of commercial fish oils AFAMPES 121 EPA (AFAMSA, Vigo, Spain) and Omega-3 RX (EnerZona, Milan, Italy) were combined to get the required EPA/DHA 1:1 ratio. Fish oil was administered by oral gavage in ω-3 PUFAs groups, using a gastric probe once a week at a dose of 0.8 mL oil/kg body weight. This oral incorporation was selected since it provides the same effect that the incorporation of fish oil onto the feed, mimics human dietary interventions and avoids oxidative degradation during feed processing. Soybean oil, obtained from cold pressing unrefined organic soybean oil, was from Clearspring Ltd. (London, UK). Soybean oil was administered by oral gavage in the STD and HFHS control groups at the same time at the same dose to compensate for the stress of probing and the excess of calories from fish oil in ω-3 PUFAs groups.

The fatty acid composition of the diets is shown in the supplementary material, **Supplementary Table S2**. HFHS diet contained about 0.05% of cholesterol, mainly from the anhydrous milk fat. Control diet can contain small amounts of cholesterol, likely minor 0.001%.

Feed intakes expressed as g/day/100g body weight and mean± standard deviation, were: STD 4.6 ± 0.5, STD+ω3 4.6 ± 0.5, HFHS 3.0 ± 0.1 and HFHS+ω3 3.2 ± 0.1. Energy intakes expressed as kcal/day/100g body weight and mean± standard deviation, were: STD 13.3 ± 1.4, STD+ω3 13.4 ± 1.3, HFHS 15.3 ± 0.5 and HFHS+ω3 15.7 ± 0.5. Energy intake is estimated as metabolizable energy based on Atwater factors, which assign 4 kcal/g to protein, 9 kcal/g to fat, and 4 kcal/g to available carbohydrates.

Rats were sacrificed by exsanguination after being intraperitoneally anesthetized with ketamine and xylazine (80 mg/kg and 10 mg/kg body weight, respectively). All the procedures followed the European Union guidelines (EU Directive 2010/63/EU) for the care and management of laboratory animals, and maximum efforts were made to minimize suffering. The pertinent permission for this specific study was obtained from the CSIC (Spanish Research Council) Subcommittee of Bioethical Issues and the regional Catalan authorities (reference number DAAM7921).

### Plasma and Tissue Sample Collection

The biological samples for fatty acids and metabolites analysis in plasma and erythrocytes were prepared according to methods previously described (21). Briefly, blood from each animal was centrifuged at 850 xg (15 min at 4°C) to remove erythrocytes (22). Then, PMSF (protease inhibitor) was added to plasma samples (erythrocyte free). Nitrogen gas was applied for remove oxygen to all plasma sample just before storing them at –80°C until used. Liver, kidney, skeletal muscle and white adipose tissue (perigonadal fat depot) were excised, washed with 0.9% NaCl solution, weighed, immediately frozen in liquid nitrogen upon the sacrifice and stored at –80°C until used.

### Plasma Biochemical Measurements

Glucose levels were measured by spotting blood on glucose strips and reading them by an enzyme electrode method using the Ascensia ELITE XL blood glucose meter (Bayer Consumer Care AG, Basel, Switzerland). Plasma insulin concentrations were measured using a Rat/Mouse Insulin ELISA kit according to the manufacturer’s instructions (Millipore Corporation, Billerica, MA, USA). These parameters have been described in the same rat cohort in previous works (23, 24).

### Biomarkers of Inflammation

Plasma IL-6 and leptin levels were quantified by Milliplex xMAP multiplex technology. The activities of alanine transaminase (AST) and aspartate transaminase (ALT) in plasma were used to test liver function, measured by Spinreact kits (Sant Esteve de Bas, Spain) and expressed as the AST/ALT ratio. These parameters have been described in the same rat cohort in previous works (20, 23).

### Lipid Content and Fatty Acids Analysis

Methods used for the analysis of lipid content in plasma, muscle and tissues were widely described in previous works (21). Briefly, a Bligh and Dyer (25) protocol was applied to plasma, liver, kidney, muscle and adipose tissue using dichloromethane:methanol:water (2:2:1, v/v) as the extraction solvent. Fatty acid composition was then analyzed through a transesterification procedure as Lepage and Roy (26) and gas chromatography (GC/FID, Clarus 500, Perkin–Elmer, MA, USA).

### Fatty Acid Desaturase (FAD) Indexes

FAD indexes as surrogate measures of desaturase activities were estimated as product-precursor ratios of individual fatty acids in plasma and adipose tissue according to Warensjö et al. (27). The desaturation from palmitic (16:0) into palmitoleic acid (16:1 ω-7) is regulated by the stearoyl-CoA desaturase SCD-16, while stearic acid (18:0) is desaturated into oleic acid (18:1 ω-9) by SCD-18. Desaturases Δ5 and Δ6 modulate the formation of ARA, EPA and DHA from LA and ALA. In detail, Δ5D regulates the desaturation from DGLA (20:3 ω-6) into ARA (20:4 ω-6). Δ6D regulates the desaturation from LA (18:2 ω-6) to GLA (18:3 ω-6) which is elongated to DGLA (20:3 ω-6). Δ6D is involved in the formation of DHA through microsomal elongation of DPA (22:5ω3) to 24:5ω3, followed by a second Δ6-desaturation step to 24:6ω3, finally β-oxidation to produce DHA. The same route works for the ω6 family, yielding 22:5ω6 from 24:5ω6 The pathway from ALA (18:3 ω-3) into EPA (20:5 ω-3) is mediated by the action of both Δ5D and Δ6D, first Δ6D desaturates ALA into SDA (18:4 ω-3), which is elongated and further desaturated by Δ5D into EPA. Therefore, FAD indexes were determined as follows: SCD-16 = [16:1 ω-7/16:0], SCD-18 = [18:1 ω-9/18:0], Δ5D = [20:4 ω-6/DGLA 20:3 ω-6], Δ6D = [20:3 ω-6/18:2 ω-6], Δ6D = [22:6 ω-3/22:5 ω-3] and Δ5/6D = [20:5 ω-3/18:3 ω-3].

### Sample Preparation by Solid Phase Extraction

Lipid mediators from adipose tissue were extracted using a modified method of a published protocol (28, 29). Frozen adipose tissue (150 mg) was cut, spiked with 12HETE-d8 (as internal standard), and extracted by sonication (1 min under 0.6 s cycle and 100% of amplitude) (Labsonic sonifier form Sartorius, Germany), in 1 mL cold-methanol containing 0.5% BHT. Samples were incubated on ice for 10 min and then centrifuged at 800 g for 10 min, at 4°C, to remove potential proteins that may cause interference. The supernatant was diluted with 4.6 mL of cold water to a final solution of methanol: water (30:70, v/v). Conditioned Oasis-HLB cartridges (60mg, 3mL, Waters, MA, USA) with 5 mL methanol (0.5% BHT) were used for purification and then, compounds were eluted with cold methyl formate (0.1% BHT) (30). Extracts were evaporated to dryness and the residue was dissolved in 30 µL cold ethanol and stored at –80°C prior to LC-MS/MS analysis.

### LC-MS/MS

Chromatographic separation was performed according to Dasilva et al. (28) in a Dionex UltiMate 3000 Series (Thermo Fisher, Rockford, IL, USA). Briefly, compounds were separated on a C18-Symmetry column, 150×2.1 mm, 3.5 μm (Waters, Milford, MA, USA) protected with a 4×2mm C18 guard cartridge provided by Phenomenex (Torrance, CA, USA). A binary eluent system of water (A) and methanol (B), both with 0.02% (v/v) of formic acid, was used as mobile phase [0-1 min (60% B), 2-12 min (80% B), 13-18 min (100% B), and 19-24 min (60% B)]. A dual-pressure linear ion trap LTQ Velos Pro (Thermo Fisher) was used for mass spectrometry analyses. Operating conditions of the ESI source were negative ion mode with a sheath gas flow rate of 40 units, spray voltage of 5.5 kV, capillary temperature of 300 °C and S-lens radio-frequency level of 60%. Nebulizing gas was nitrogen and collision gas was helium. Instrument control and data acquisition were done with Xcalibur software. According to the *m/z* of their parent ion, compounds were classified into 22 groups. Data acquisition was a full scan mode ranged from 90 to 400 m/z units with the 22 parent masses in a single segment during the run. LOD and LOQ values ranged between 0.01 to 17.65 ng/mL and 0.03 to 58.84 ng/mL, respectively as previously described (28).

### Statistics

A two-way analysis of variance (ANOVA) was run with R free software (version 3.2.4) on a sample of 36 rats to examine the effect of the background diet (STD or HFHS) and the supplement (control or ω3-PUFAs) on each dependent variable reported in tables. Normal distribution and heterogeneity were evaluated and non-parametric Kruskal Wallis analyses were required when data distribution did not fit a Gaussian model or heterogeneity was found in variances. In the two-way ANOVA analysis, significant differences (p < 0.05) were expressed using different superscripts: *p < 0.05 significant differences given by the factor “diet” (STD vs. HFHS); $ p < 0.05 significant differences given by the factor “supplement” (control vs ω-3). If the two-way ANOVA determines a significant interaction (p < 0.05) between the effects of background diet and supplement on a certain dependent variable, the interaction is reported by using the superscript #. When significant differences were found in the factors, the post-hoc Fisher least square difference (Fisher LSD) pairwise test was run to compare means values. Means with different (a, b, c, d) superscript indicate significant differences (p < 0.05). Data presented are expressed as mean ± SEM or SD.

## Results and Discussion

### Insulin Resistance, Ectopic Lipid Deposition and Low-Grade Inflammation

Rats fed HFHS diets for 21 weeks developed a general prediabetic state. That state was associated with higher plasma insulin levels to maintain glucose levels into the normal range, and a significant increase of the perigonadal white adipose tissue as described in previous works (23, 31). Accordingly, the HFHS diet provoked a significant increment of plasma insulin (**Table 1**). Indeed, rats fed HFHS diet almost doubled their plasma insulin levels as compared with STD-fed rats. Plasma glucose levels tendered to be slightly higher in the HFHS group at the end of the nutritional intervention and the supplementation with ω-3 PUFAs did not affect them in both STD and HFHS groups. However, rats fed HFHS diet supplemented with ω-3 PUFAs showed a trend towards lower plasma insulin values than their corresponding HFHS-fed counterparts; and plasma insulin levels of the HFHS+ ω-3 group were not significantly different from the STD control group by the end of nutritional intervention. Thereby, 67% of the rats fed HFHS diet supplemented with ω-3 PUFAs showed lower levels of insulin than those achieved by the HFHS control group. In addition, 56% of the HFHS fed rats supplemented with fish oil reduced the levels of plasma glucose.

**Table 1:** Morphological values, lipid content, insulin resistance and inflammatory parameters from Sprague-Dawley rats with different diets.

	STD		STD+ω3		HFHS		HFHS+ω3	
	Mean	SD	Mean	SD	Mean	SD	Mean	SD
Body weight after 21 weeks feeding^1^	526.25 ^a^	31.16	522.78 ^a^	39.40	568.33 ^a^	24.25	579.89 ^a^	35.66
Perigonadal Adipose Tissue^1^*	8.99 ^a^	3.16	8.53 ^a^	2.52	13.12 ^b^	3.92	13.28 ^b^	4.41
Adiposity index (%)^1^*	1.67 ^a^	0.44	1.65 ^a^	0.38	2.37 ^b^	0.75	2.32 ^b^	0.69
% FAT CONTENT Erythrocytes*	2.09 ^a^	0.24	2.04 ^a^	0.16	2.27 ^b^	0.16	2.21 ^b^	0.21
% FAT CONTENT Plasma*^$#^	3.91 ^a^	0.75	3.96 ^a^	1.28	4.34 ^ab^	1.37	2.94 ^b^	0.84
% FAT CONTENT Liver^$^	7.01 ^a^	0.51	6.36 ^b^	0.28	7.34 ^a^	0.45	6.55 ^b^	0.38
% FAT CONTENT Kidney*^$^	4.31 ^a^	0.23	4.11 ^b^	0.24	5.65 ^c^	0.56	4.39 ^a^	0.39
% FAT CONTENT Muscle*^$^	2.51 ^a^	0.71	2.37 ^b^	0.59	3.14 ^c^	0.99	2.51 ^a^	0.31
% FAT CONTENT Adipose Tissue*^$^	96.32 ^a^	2.05	92.73 ^b^	3.16	99.51 ^c^	2.03	97.01 ^a^	1.20
Plasma Total Fatty Acids mg/mL*^$^	2.19 ^a^	0.25	1.96 ^ab^	0.31	1.85 ^ab^	0.21	1.70 ^b^	0.36
Plasma Insulin ng/ml^1^*	0.56 ^a^	0.32	0.65 ^a^	0.19	1.81 ^b^	0.82	1.46 ^b^	0.72
Plasma Glucose mg/ml^1^	63.00 ^a^	4.84	63.44 ^a^	4.10	70.78^a^	4.99	71.33 ^a^	5.32
AST/ALT^1^*^$#^	2.64 ^a^	0.52	2.89 ^a^	0.9	3.14 ^b^	0.92	2.37 ^a^	0.29
IL-6 pg/mL^1^*^$#^	43.60 ^a^	24.4	47.41 ^a^	8.41	47.50 ^b^	11.01	39.3 ^a^	6.5
Leptin [pg mL−1] ^1^	1104	59.7			2200.1	98.6	1792.3	65.8

^1^These parameters have partially been published in a previous report (20, 23). Adiposity index: (total abdominal fat×100)/body weight. Hepatosomatic index: (liver weight×100)/body weight.

Two-way ANOVA analyses were done. *p < 0.05 significant differences given by the factor “diet” (STD vs. HFHS); ^$^ p < 0.05 significant differences given by the factor “supplement” (control vs ω-3). Superscript ^#^ indicates significant interaction (p < 0.05) between the factors diet (ST and HFHS) and supplement (control and ω-3 PUFAs supplement). Means with different superscript ^(a,b,c,d)^indicate significant differences (p < 0.05) (analyzed by post-hoc Fisher LSD).

Values with different superscript letters in the same row indicate significant difference at p < 0.05 between dietary groups (n=9 per group).

High fat and sucrose diets promoted insulin resistance and impaired glucose tolerance through low-grade systemic inflammation associated with liver inflammatory cell infiltration, increased levels of plasma IL-6, PGE2, and reduced levels of protective short-chain fatty acids (23). Even if no significant differences were observed in either final body weight among the groups, consumption of HFHS diets enlarged the perigonadal white adipose tissue content after 21 weeks and significantly increased the adiposity index as compared to STD control (almost 35%) (23). Interestingly, ectopic lipid deposition was also observed from the values of fat content in different organs and tissues corresponding to HFHS- fed rats (**Table 1**). The high energetic diet resulted in a general increment of the lipid content of plasma and tissues, and such increment was significant for kidney, skeletal muscle and adipose tissue.

Supplementation with ω-3 PUFAs in both STD and HFHS groups did not provoke significant differences for the body weight, perigonadal adipose tissue and the adiposity index. However, STD or high energetic diets supplemented with ω-3 PUFAs significantly diminished lipid content in all the studied tissues and organs (**Table 1**). Such reduction was especially relevant for HFHS-fed rats supplemented with ω-3 PUFAs which showed general fat content in plasma, liver, kidney, skeletal muscle and adipose tissue similar to the STD rats. These findings are in agreement with the histological liver steatosis as we previously reported in the same cohort of rats (24).

Regarding biomarkers of inflammation, as it has been previously reported, this experimental high-fat and sucrose dietary model induced a state of systemic low-grade inflammation in male Sprague-Dawley rats evidenced from their altered microbiota, liver lobular inflammation and plasma levels of IL-6 and leptin (23). Accordingly, rats fed HFHS have evidenced higher levels of plasma IL-6 and leptin than their counteracted rats fed STD diets. HFHS-fed rats also presented lobular liver inflammation with lymphoplasmacytic inflammatory infiltration around the blood vessels (23). Finally, HFHS-fed rats resulted in an altered microbiota with a reduced Bacteroidetes : Firmicutes ratio and increased proportions of Enterobacteriales with as compared to the STD group (23). This experimental prediabetic model based on high-fat and sucrose feeding induced a state of systemic low-grade inflammation in other strains of rats (31).

Supplementation with ω-3 PUFAs of both STD and HFHS groups showed a tendency to reduce the increased levels of plasma IL-6 and leptin found in HFHS-fed rats (**Table 1**). Additionally, ω-3 PUFAs partially counteracted liver lobular inflammation as reported previously (23) and decreased the marker of hepatic injury, AST/ALT ratio (**Table 1**).

### Lipid Profiling

The fatty acid composition of adipose tissue largely reflected the profile of diet fatty acids (**Supplementary Table S2**). Linoleic acid was the main fatty acid in the adipose tissue of rats fed the STD diet (~40% of total fatty acids), followed by oleic and palmitic acids **(****Table 2****)**. The other fatty acids basically maintained their dietary proportions, with minor increments in saturated fatty acids and their monounsaturated derivatives. The adipose tissue of HFHS-fed rats reproduced the proportions of diet fatty acids as well, although some differences were observed. As previously described in other rodent models (19), adipose tissue of HFHS-fed rats concentrated notable levels of oleic acid, which became the major component in the tissue. Interestingly, palmitoleic acid was also found to increase. Accumulation of oleic and palmitoleic acid in adipose tissue has been linked to obesity (32), hypertriacylglycerolemia, and the risk of developing insulin resistance (33). This association might be strengthened under high carbohydrate intake (34).

**Table 2:** Fatty acid composition of the adipose tissue from animals supplemented with STD and HFHS diets divided in controls and ω-3 PUFAs supplemented groups.

	STD		STD-+ω3		HFHS		HFHS+ω3	
FATTY ACID	Mean	SD	Mean	SD	Mean	SD	Mean	SD
14:0*^$^	1.06^a^	0.07	1.19^b^	0.09	6.19^c^	0.28	6.05 ^c^	0.18
16:0*	21.23^a^	0.70	20.90^a^	1.01	27.37^b^	0.81	27.99^b^	0.63
16:1ω7*	3.95^a^	0.78	4.64^a^	2.37	6.31^b^	1.08	5.73^b^	0.56
18:00*	2.26^a^	0.26	2.25^a^	0.36	4.12^b^	0.41	4.35^b^	0.31
18:1ω9*	21.95^a^	0.52	22.09^a^	0.66	39.02^b^	1.13	39.50^b^	0.93
18:1ω7*	4.85^a^	0.31	4.82^a^	0.32	3.64^b^	0.25	3.88^b^	0.07
18:2ω6*^$^	39.79^a^	1.35	38.39^a^	2.36	9.46^a^	0.26	8.57^b^	0.25
18:3ω3*	1.60^a^	0.08	1.62^a^	0.08	0.66^b^	0.07	0.61^b^	0.07
20:3ω6*	0.14^a^	0.02	0.16^a^	0.04	0.00^b^	0.00	0.00^b^	0.00
20:4ω6*	0.66^a^	0.10	0.64^a^	0.31	0.13^b^	0.05	0.12^b^	0.01
**20:5ω3**^$#^	<0.01^a^	0.00	0.13^b^	0.02	<0.01^a^	0.00	<0.01^a^	0.00
22:4ω6*	0.18^a^	0.11	0.21^a^	0.07	0.00^a^	0.00	0.00^a^	0.00
22:5ω3*^$^	0.17^a^	0.03	0.36^b^	0.04	<0.01^a^	0.00	0.06^b^	0.03
22:6ω3*^$^	0.12^a^	0.03	0.50^b^	0.03	<0.01^a^	0.00	0.09^b^	0.02
ω3*^$^	2.19 ^a^	0.07	2.96^b^	0.25	0.81^c^	0.06	0.93^d^	0.10
ω6*^$^	41.15 ^ab^	1.26	39.78^b^	1.83	9.70 ^c^	0.30	8.69^d^	0.25
ω6/ω3*^$^	18.79 ^a^	1.15	13.42 ^b^	0.86	11.98 ^b^	0.74	9.34 ^c^	0.50

Two-way ANOVA analyses were done. *p < 0.05 significant differences given by the factor “diet” (STD vs. HFHS); ^$^ p < 0.05 significant differences given by the factor “supplement” (control vs ω-3). Superscript ^#^ indicates significant interaction (p < 0.05) between the factors diet (ST and HFHS) and supplement (control and ω-3 PUFAs supplement). Means with different superscript ^(a,b,c,d)^indicate significant differences (p < 0.05) (analyzed by post-hoc Fisher LSD).

Results are expressed as percentage of total fatty acids (mg/100mg of total fatty acids). Results are expressed as means and standard deviation (SD). Values with different superscript letters in the same row indicate significant difference at p < 0.05 between dietary groups (n=9 per group).

Supplementation with ω-3 PUFAs of STD-fed rats increased the levels of saturated fatty acids, myristic and palmitic acids, EPA and DHA while decreasing ARA, oleic and linoleic acids. Then, ω-6/ω-3 ratio found in adipose tissue was significantly reduced in the ω-3-supplemented group as compared to the control. In HFHS-fed rats, the levels of myristic acid, EPA and DHA increased with the ω-3 supplementation as compared to the control group with a subsequent decrease of oleic acid, ARA and linoleic acid. The inflammatory index ω-6/ω-3 was significantly reduced in the HFHS+ω-3 rats as well.

Fatty acid desaturase indexes (FADS) evaluated as product:precursor ratios in adipose tissue were compared with those in plasma (27). Results are shown in **Table 3**. The high energy diet resulted in an increment of plasma stearoyl-CoA desaturase 1 indexes: SCD-16 [palmitoleic/palmitic] and SCD-18 [oleic/stearic]. This increase was also found in adipose tissue which showed a significantly higher value of SCD-16 in HFHS-fed rats than in STD-fed rats. Increased SCD-1 activities are associated with augmented adiposity and progression of the obesity syndrome in humans (35, 36). Experimental animal studies have also revealed the association between SCD-1 and obesity and insulin resistance (37). More SCD-1 activity has demonstrated to favor the storage of fat (37).

**Table 3:** FAD indexes from total fatty acid data of plasma and white adipose tissue calculated as product/precursor ratio.

	STD		STD+ω3		HFHS		HFHS+ω3	
**DESATURASES IN PLASMA**	**Mean**	**SD**	**Mean**	**SD**	**Mean**	**SD**	**Mean**	**SD**
SCD-16 = [palmitoleic (16:1ω7)/palmític (16:0)] *	0.068 ^a^	0.01	0.068 ^a^	0.01	0.078 ^b^	0.01	0.109 ^b^	0.04
SCD-18 = [oleic (18:1ω9)/estearic (18:0)] *	0.859 ^a^	0.13	0.837 ^a^	0.13	1.597 ^b^	0.30	1.589 ^b^	0.36
Δ5D = [ARA (20:4ω6)/DGLA (20:3ω6)] *^$^	123.536 ^a^	11.12	70.814 ^b^	12.92	48.031 ^bc^	17.08	31.760 ^c^	3.17
Δ6D = [DGLA (20:3ω6)/LA (18:2ω6)] *^$^	0.013 ^a^	0.00	0.018 ^b^	0.01	0.053 ^c^	0.01	0.058 ^c^	0.01
Δ6D = [DHA (22:6ω3)/DPA (22:5ω3)] *^$#^	2.948 ^a^	0.38	2.656 ^a^	0.43	4.235 ^b^	1.01	5.808 ^c^	0.52
Δ5D + Δ6D = [EPA (20:5ω3)/ALA (18:3ω3)] ^$^	1.854 ^a^	0.52	2.963 ^b^	0.77	2.308 ^ab^	0.48	6.157 ^c^	0.87
**DESATURASES IN ADIPOSE TISSUE**	**Mean**	**SD**	**Mean**	**SD**	**Mean**	**SD**	**Mean**	**SD**
SCD-16 = [palmitoleic (16:1ω7)/palmític (16:0)] ^$#^	0.186 ^a^	0.03	0.227 ^ab^	0.13	0.224 ^b^	0.04	0.204 ^a^	0.01
SCD-18 = [oleic (18:1ω9)/estearic (18:0)]	9.928 ^a^	1.13	10.201 ^a^	2.21	9.471 ^a^	0.19	9.081 ^a^	0.39
Δ5D = [ARA (20:4ω6)/DGLA (20:3ω6) *]	4.999 ^a^	0.66	3.997 ^a^	0.86	No detected	–	No detected	No detected
Δ6D = [DHA (22:6ω3)/DPA (22:5ω3)] ^$^	0.755 ^a^	0.13	1.386 ^b^	0.13	No detected	–	1.512 ^c^	0.33
Δ6D = [DGLA (20:3ω6)/LA (18:2ω6)] *	0.003 ^a^	0.00	0.004 ^a^	0	0.000 ^b^	0.00	0.000 ^b^	0.00
Δ5D + Δ6D = [EPA (20:5ω3)/ALA (18:3ω3)]	0.000 ^a^	0.00	0.076 ^b^	0.05	0.000 ^a^	0.00	0.000 ^a^	0.00

Two-way ANOVA analyses were done. *p < 0.05 significant differences given by the factor “diet” (STD vs. HFHS); ^$^ p < 0.05 significant differences given by the factor “supplement” (control vs ω-3). Superscript ^#^ indicates significant interaction (p < 0.05) between the factors diet (ST and HFHS) and supplement (control and ω-3 PUFAs supplement). Means with different superscript ^(a,b,c,d)^indicate significant differences (p < 0.05) (analyzed by post-hoc Fisher LSD).

Results are expressed as means and standard deviation (SD). Values with different superscript letters in the same row indicate significant difference at p < 0.05 between dietary groups (n=9 per group).

Additionally, the consumption of HFHS diets revealed an increment of plasma Δ6D = [DGLA (20:3 ω-6)/LA (18:2 ω-6)] together with a decrease of plasma Δ5D = [ARA (20:4 ω-6)/DGLA (20:3 ω-6)] activities, leading to the accumulation of DGLA. Finally, an up-regulation of plasma Δ6D related to the production of DHA (22:6 ω-3) *via* DPA (22:5 ω-3)/24:5ω3 and 24:6ω3 as described above to accumulate more DHA, was also observed. These FAD indexes, Δ5D and Δ6D, remained unchanged in adipose tissue of HFHS-fed rats compared to STD controls.

Interestingly, ω-3 PUFAs supplementation modulated the FAD indexes in both STD- and HFHS-fed rats. Plasma desaturases indexes involving PUFAs were generally modified and no changes on SCD-1 related to the synthesis of monounsaturated fatty acids were observed. Adipose instead, tissue demonstrated a significant modulation of SCD-1 indexes (**Table 3**). In consequence, supplementation with ω-3 PUFAs reduced SCD-16 and SCD-18 indexes in prediabetic adipose tissue. When they were added to the HFHS context, which is more prone to accumulate oleic and palmitic acids, ω-3 PUFAs were effective at modulating SCD-1 indicating a potential reduction of the synthesis *de novo* mediated by this desaturase. These results are in agreement with previous results found in plasma and liver of Wistar rats fed HFHS diets (19). Reduction of SCD-1 in murine adipose tissue has been related to decreased triglycerides levels in 3T3-L1 adipocytes, and altered markers of fatty acid reesterification, glyceroneogenesis, and lipolysis (38). So the lesser SCD-1 indexes found in white adipose tissue of HFHS-fed rats supplemented with ω-3 PUFAs might be associated with the lesser fat content measured in adipose tissue of these animals (**Table 1**). Moreover, in some cell types, such as adipocytes, β-cells, endothelial cells, macrophages, and myocytes, SCD-1 participates in the regulation of inflammation and stress (39).

As regards to PUFAs synthesis, both plasma and adipose tissue desaturase indexes demonstrated that HFHS-fed rats supplemented with ω-3 PUFAs showed significantly higher indexes of Δ6D related to the production of DHA *via* DPA/24:5ω-3/24:6 ω-3. However, this result could be attributed to the amounts of 22:6 ω-3 and 22:5 ω-3 provided by the feeds (**Supplementary Table S2**). And both dietary frameworks showed significantly higher values of plasma Δ5D/Δ6D = [20:5 ω-3/18:3 ω-3] for STD- and HFHS-fed rats supplemented with ω-3 PUFAs according to the fatty acids provided by feeds as well. Adipose tissue of STD rats showed the same result.

Supplementation with ω-3 PUFAs led to lower values of Δ5D = [20:4 ω-6/20:3 ω-6] in plasma for STD- and HFHS-fed rats. This result was in agreement with the amounts of 20:3 ω-6 and ARA incorporated through the fish oil supplement to the feeds. The higher amount of 20:4 ω-6 than 20:3 ω-6 in the control feeds implied higher Δ5D index in the control groups than in the ω-3 PUFAs supplemented groups. Finally, there were no effects for Δ6D = [DGLA (20:3 ω-6)/LA (18:2 ω-6)] accordingly with the amounts of 20:3 ω-6 and 18:2 ω-6 provided by the feeds as well. According to several studies, deficiency of SCD-1 provides positive metabolic effects and reduce obesity-associated to adipose tissue inflammation (37). However, other desaturases as Δ5D or Δ6D have not been clearly associated with these metabolic changes.

### Lipid Mediators and Specialized Resolvers of Inflammation

**Table 4** showed the levels of lipid mediators found in the perigonadal white adipose tissue of the STD and HFHS rats. The effect of the supplementation with ω-3 PUFAs in both dietary frameworks is also shown in **Table 4**. The aim was to identify and quantify through a LC-MS/MS-based metabolo-lipidomic platform the pathway-oriented profiling of lipid mediators derived from ω-3 and ω-6 PUFAs together with SPMs, namely lipoxins, RVs, PDs, and MaRs as well as their intermediate monohydroxy biosynthetic pathway markers of RvD1, PD1 and PDX (17HDHA), RvE1 (11HEPE and 18HEPE), and MaR1 (14HDHA) (9).

**Table 4:** Levels of lipid mediators in white adipose tissue derived from ARA, EPA and DHA.

		STD		STD + ω3		HFHS		HFHS + ω3	
**ARA DERIVATIVES**									
**Lipid Mediator**	**Pathway**	**Mean**	**SEM**	**Mean**	**SEM**	**Mean**	**SEM**	**Mean**	**SEM**
5HETE*^$^	5LOX/GPX	28.36 ^a^	1.13	18.21 ^b^	1.27	9.72 ^c^	1.26	11.91 ^c^	1.26
5OxoETE*	5LOX/DHO	9.44 ^a^	0.08	8.71 ^a^	0.14	8.51 ^a^	0.07	8.80 ^a^	0.10
11HETE*^$^**#**	Non enzy	39.95 ^a^	1.18	18.92 ^b^	2.88	18.59 ^b^	2.90	17.38 ^b^	2.08
12HETE*^$^**#**	12LOX/GPX	67.88 ^a^	1.51	41.84 ^b^	4.58	34.99 ^b^	5.43	26.56 ^c^	2.43
12OxoETE*^$^**#**	12LOX/DHO	39.17 ^a^	0.47	39.98 ^a^	2.77	37.51 ^b^	1.79	40.00 ^a^	0.74
15HETE*^$^	15LOX/GPX	36.78 ^a^	1.20	17.72 ^b^	1.60	18.65 ^b^	2.59	18.50 ^b^	1.86
20HETE*^$^**#**	CYP	2.70 ^a^	0.07	1.95 ^b^	0.29	2.96^c^	0.07	0.00 ^d^	0.00
(±)5(6)-EET*#	CYP	23.95 ^a^	0.36	18.71 ^b^	1.52	10.56 ^c^	0.86	13.77 ^c^	0.50
**(±)5(6)-DiHET**	CYP/sEH	0.00 ^a^	0.00	0.00 ^a^	0.00	0.00 ^a^	0.00	0.00 ^a^	0.00
(±)8(9)-EET*	CYP	8.49 ^a^	0.14	8.09 ^a^	0.35	6.50 ^b^	0.22	6.78 ^b^	0.13
**(±)8(9)-DiHET**	CYP/sEH	0.00 ^a^	0.00	0.00 ^a^	0.00	0.00 ^a^	0.00	0.00 ^a^	0.00
(±)11(12)-DiHET*	CYP/sEH	0.06 ^a^	0.00	0.05 ^a^	0.01	0.00 ^b^	0.00	0.00 ^b^	0.00
**(±)14(15)-EET**	CYP	1.66 ^a^	0.14	1.27 ^a^	0.31	1.66 ^a^	0.26	1.14 ^a^	0.26
(±)14(15)-DiHET*^$^**#**	CYP/sEH	0.35 ^a^	0.04	0.25 ^b^	0.05	0.52 ^c^	0.03	0.00 ^d^	0.00
PGE2*^$^	COX	182.51 ^a^	6.30	142.03 ^b^	18.90	164.61 ^a^	19.37	148.44 ^ab^	9.81
PGD2*^$^**#**	COX*#	42.47 ^a^	1.91	62.19 ^b^	10.26	56.67 ^b^	3.75	51.63 ^ab^	5.43
PGD2/PGE2*^$^**#**	COX	0.23 ^a^	0.03	0.44 ^b^	0.18	0.34 ^ab^	0.06	0.35 ^ab^	0.18
**LTB4**	5LOX	0.00 ^a^	0.00	0.00 ^a^	0.00	0.00 ^a^	0.00	0.00 ^a^	0.00
**EPA DERIVATIVES**									
**Lipid Mediator**	**Pathway**	**Mean**	**SEM**	**Mean**	**SEM**	**Mean**	**SEM**	**Mean**	**SEM**
5HEPE*^$^**#**	5LOX/GPX	1.54 ^a^	0.24	4.18 ^b^	0.24	0.00 ^c^	0.00	0.00 ^c^	0.00
**11HEPE**^$^**#**	Non enzy	2.15 ^a^	0.38	3.72 ^b^	0.27	1.84 ^a^	0.27	3.27 ^b^	0.25
**12HpEPE**^$^**#**	12LOX	22.43 ^a^	3.32	62.30 ^b^	17.53	19.68 ^a^	5.68	19.98 ^a^	2.80
**12HEPE**^$^**#**	12LOX/GPX	3.99 ^a^	0.61	12.13 ^b^	1.20	1.97 ^a^	0.44	6.84 ^b^	0.78
15HpEPE*#	15LOX	8.46 ^a^	0.73	8.71 ^a^	0.64	5.16 ^a^	0.03	4.69 ^a^	0.56
**15HEPE**^$^	15LOX/GPX	4.66 ^a^	0.81	7.09 ^b^	0.57	3.96 ^a^	0.39	6.34 ^b^	0.51
18HEPE*^$^**#**	Non enzy	4.64 ^a^	0.27	5.66 ^a^	0.63	2.43 ^a^	0.09	9.04 ^b^	0.68
**(±)17(18)-DiHETE**	CYP/sEH	0.00 ^a^	0.00	0.00 ^a^	0.00	0.00 ^a^	0.00	0.00 ^a^	0.00
**DHA DERIVATIVES**									
**Lipid Mediator**	**Pathway**	**Mean**	**SEM**	**Mean**	**SEM**	**Mean**	**SEM**	**Mean**	**SEM**
4HDoHE*^$^	5LOX/GPX	3.17 ^ab^	0.27	3.57 ^b^	0.20	2.44 ^a^	0.07	3.75 ^b^	0.30
11HDoHE*^$^	12LOX/GPX	1.52 ^a^	0.23	2.68 ^b^	0.15	1.06 ^c^	0.19	2.94 ^b^	0.34
**14HDoHE**^$^	12LOX/GPX	20.85 ^a^	2.72	27.96 ^b^	0.34	18.60 ^a^	2.69	28.75 ^b^	2.36
**17HDoHE**^$^**#**	15LOX/GPX	24.46 ^a^	3.20	33.54 ^b^	2.26	29.78 ^a^	2.51	37.63 ^b^	1.85
PDX*^$^**#**	15LOX/GPX	1.31 ^a^	0.32	0.99 ^a^	0.00	0.22 ^b^	0.03	1.34 ^a^	0.15
**(±)7(8)-EDP**	CYP	3.76 ^a^	0.10	4.62 ^b^	0.24	4.01 ^a^	0.28	4.64 ^ab^	0.26
(±)10(11)-EDP*^$^	CYP	2.45 ^a^	0.11	2.81 ^b^	0.09	1.95 ^a^	0.14	2.54 ^b^	0.18
(±)13(14)-EDP*^$^	CYP	1.22 ^a^	0.07	1.75 ^b^	0.08	1.09 ^a^	0.10	1.45 ^b^	0.11
(±)19(20)-EDP*	CYP	5.20 ^a^	0.42	5.86 ^ab^	0.30	0.00 ^b^	0.00	6.60 ^c^	0.51
(±)19(20)-DiHDPA*#	CYP/sEH	3.14 ^a^	0.06	3.73 ^a^	0.24	0.00 ^b^	0.00	0.00 ^b^	0.00

Two-way ANOVA analyses were done. *p < 0.05 significant differences given by the factor “diet” (STD vs. HFHS); ^$^ p < 0.05 significant differences given by the factor “supplement” (control vs ω-3). Superscript ^#^ indicates significant interaction (p <0.05) between the factors diet (ST and HFHS) and supplement (control and ω-3 PUFAs supplement). Means with different superscript ^(a,b,c,d)^indicate significant differences (p < 0.05) (analyzed by post-hoc Fisher LSD).

Results are expressed as ng/mL. Results are expressed as means with their standard errors of the mean (SEM); n=9 per group.

#### Lipid Mediator Profile of Adipose Tissue of STD- vs. HFHS-Fed Rats

Consumption of HFHS diet resulted in a lower concentration of lipid mediators in adipose tissue. Therefore, the concentration in prediabetic rats fed the HFHS diet was 90 ng/g vs. 120 ng/g in STD-fed rats. This fact was especially due to a decrease in eicosanoids and docosanoids derived from long-chain PUFAs as ARA, EPA and DHA which were found in lower concentrations in HFHS-fed rats (**Table 2**).

A detailed analysis of the type and level of eicosanoids and docosanoids formed in adipose tissue provided a plethora of intrinsic signals showing an inflammatory response in prediabetic adipose tissue. Therefore, reduced levels of epoxides and oxo-derivatives together with increasing concentrations of di-hydroxides and pro-inflammatory PGD2 and a reduction of protectin PDX were observed in rats fed the HFHS diet. This adipose inflammatory state agreed with the low-grade systemic inflammation previously evidenced from their altered microbiota (23), liver inflammation (23) and levels of plasma IL-6, leptin and the ratio ASL/ALT (**Table 1**).

Accordingly, **Table 4** shows a significant reduction of epoxides generated *via* cytochrome P450 (CYP) activity from ARA, namely (±)5(6)-EET and (±)8(9)-EET, and for DHA as (±)10(11)-EDP and (±)19(20)-EDP. Epoxides have been described for their anti-inflammatory properties and their beneficial effect in decreasing insulin resistance, both effects mediated through the activation of PPARgamma (40). Keto-lipid mediators derived from the 5LOX activity on ARA, as 5oxoETE, and from the 12LOX, as 12oxoETE, were found decreased in prediabetic adipose tissue as well. These keto-compounds, being not well-known derivatives yet, have been associated with an activation of the PI3-Akt pathway and therefore enhanced insulin sensitivity (41). The decrease of both epoxides and oxo-derivatives in adipose tissue of HFHS-fed rats appeared to contribute to their lower insulin sensitivity, which corresponded to the hyperinsulinemia measured in these rats (**Table 1**).

As a consequence of the reduction in epoxides, HFHS consumption provoked an increase in the formation of some pro-inflammatory di-hydroxides derived from the enzymatic action of epoxyhidrolase, sEH, on their corresponding ARA-epoxides. Higher synthesis of (±)14(15)-DiHET derived from (±)14(15)-EET in adipose tissue of prediabetic HFHS-fed rats than in STD was detected. In consequence, the balance (±)14(15)-DiHET/(±)14(15)-EET was 0.31 in HFHS-fed rats vs. 0.21 in STD-fed rats. This finding indicated more activity of the enzyme sEH in rats fed the hyperenergetic diet likely related to an incipient inflammatory status of the adipose tissue. Tissue levels of CYP epoxides derived from PUFAs are limited by sEH activity since that enzyme converts these anti-inflammatory mediators into less active diols. These diol-containing DHETs have drastically reduced biologic activity (42) and those derived from ARA have been described as highly active pro-inflammatory and oxidative stress-inducing substances (40). Hence, many studies have addressed the inhibition of sEH to discover sEH inhibitors which can efficiently increase the longevity of anti-inflammatory EETs (43). sEH appeared to be more active on ARA rather than on other PUFAs. Accordingly, other di-hydroxy lipids were found decreased in prediabetic rats fed the HFHS diet as the scarcely pro-inflammatory and pro-oxidant (±)19(20)-DiHDPA derived from DHA.

HFHS consumption significantly modified COX derived lipid mediators as prostaglandins resulting from ARA: PGE2 and PGD2. In line with some previous works in obese adipose tissue, PGD2 showed substantial up-regulation (44). Also in line with previous studies that addressed diet-induced obesity, PGE2 showed a significant down-regulation. The differential expression of COX within cells at sites of inflammation determines the profile of prostanoid production. Previous results have suggested that PGE2 normally plays a key role in regulating anti-inflammation and immune suppression during lipolysis (45). PGE2 acutely recruits adipose tissue macrophages mostly in the anti-inflammatory M2 state, which would likely restrict the local lipid concentration and lipotoxicity. In agreement with results found for prediabetic rats fed HFHS diet, a more limited PGE2 production during the development of diet-induced prediabetes might play a role in the activation of inflammatory immune cells. Additionally, PGE2 has induced expression of brown markers in white adipose tissue addressing adipocyte trans-differentiation towards beige/brite cells and exhibiting anti-inflammatory actions (44).

The pro- or anti-inflammatory behavior of PGD2 depends on the disease process and etiology. PGD2 has been described as responsible for driving macrophages toward an M2 phenotype (44). In line with a potential anti-inflammatory role, PGD2 level in adipose tissue macrophages was positively correlated with both peripheral and adipose tissue insulin sensitivity in humans (44). However, high-fat feeding studies in mice have demonstrated that overproduction of PGD2 *in vivo* lead to pronounced adipogenesis, and increased insulin sensitivity (46). Accordingly, the higher concentration of PGD2 in prediabetic adipose tissue of these HFHS-fed rats can be associated with the ectopic lipid deposition found (**Table 1**) and adipocyte hypertrophy detected (31).

**Figure 1:**
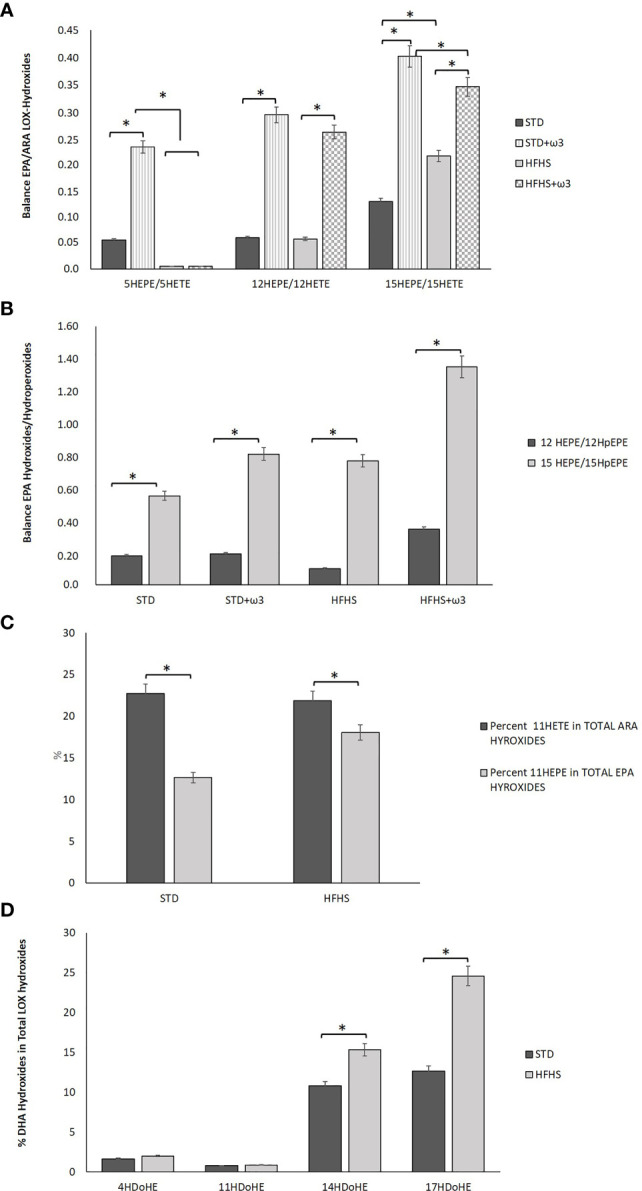
Comparison of lipid mediators formed in the perigonal adipose tissue of rats fed STD, STD+ω3, HFHS or HFHS+ω3 diets. **(A)**. Balance of several EPA/ARA LOX-Hydroxides, particularly, 5HEPE/5HETE, 12HEPE/12HETE and 15HEPE/15HETE measured in adipose tissue from rats fed STD, STD+ω3, HFHS or HFHS+ω3 diets. **(B)**. Balance of several Hydroxides/Hydroperoxides from EPA, particularly 12HEPE/12HpHEPE and 15HEPE/15HpHEPE, measured in in adipose tissue from rats fed STD, STD+ω3, HFHS or HFHS+ω3 diets. **(C)**. Percentage of 11HETE in total ARA Hydroxides and percentage of 11HEPE in total EPA Hydroxides measured in adipose tissue from rats fed STD or HFHS diets. **(D)**. Percentage of several DHA Hydroxides, particularly 4HDoHE, 11HDoHE, 14HDoHE and17HDoHE, in total LOX-Hydroxides measured in adipose tissue from rats fed STD or HFHS diets (n=9 per group). *Significant differences among groups (p < 0.05).

The study of the hydroxides derived from lipoxygenases (LOXs) activity upon ARA and EPA revealed an interesting feature regarding adipose tissue homeostasis in prediabetic conditions. To counteract the inflammatory state described above, a compensatory mechanism aimed to favor the formation of EPA and DHA metabolites over ARA seemed to be activated in adipose tissue of HFHS-fed rats. Therefore, although the proportions of ARA in adipose tissue were similar in STD versus HFHS-fed rats (**Table 2**) and the net concentration of hydroxides was lower in HFHS fed animals, the formation of ARA derived-hydroxides was significantly inhibited in prediabetic rats (**Table 5**). Then, the ratio EPA-hydroxides/ARA-hydroxides was favored in HFHS. In particular, in spite of the fact that the relative proportion of ARA was higher than the EPA one in adipose tissue, the formation of 15HEPE was favored over the formation of 15HETE in prediabetic rats (**Figure 1A**). The balance 15HEPE/15HETE was 0.13 for STD rats and 0.21 for HFHS rats. Therefore, white adipose tissue of rats fed the HFHS diet enhanced the formation of less inflammatory EPA-derived monohydroxides over the formation of high pro-inflammatory ARA monohydroxides.

**Table 5:** Percent distribution of ARA, EPA and DHA in total PUFAs present in adipose tissue.

	STD		STD+ω3		HFHS		HFHS+ω3	
	Mean	SD	Mean	SD	Mean	SD	Mean	SD
% ARA^$^	74.05 ^a^	1.21	52.30 ^b^	0.98	73.75 ^a^	2.07	50.79 ^b^	1.53
% EPA*^$#^	6.30 ^a^	0.88	14.71 ^b^	1.43	4.41 ^c^	0.39	12.50 ^c^	0.86
% DHA^$^	19.64 ^a^	0.59	32.98 ^b^	1.22	21.82 ^a^	1.01	36.73 ^b^	1.25
% ARA hydroxides in total hydroxides*^$#^	72.39 ^a^	2.12	54.00 ^b^	1.62	57.76 ^b^	1.74	42.99 ^c^	2.14
% EPA hydroxides in total hydroxides^$#^	6.99 ^a^	0.17	8.90 ^b^	1.07	6.93 ^a^	0.22	14.74 ^c^	0.23
% DHA hydroxides in total hydroxides*^$#^	20.60 ^a^	1.44	37.09 ^b^	1.17	35.29 ^b^	0.63	42.25 ^c^	1.97

Two-way ANOVA analyses were done. *p < 0.05 significant differences given by the factor “diet” (STD vs. HFHS); ^$^ p < 0.05 significant differences given by the factor “supplement” (control vs ω-3). Superscript ^#^ indicates significant interaction (p < 0.05) between the factors diet (ST and HFHS) and supplement (control and ω-3 PUFAs supplement). Means with different superscript ^(a,b,c,d)^indicate significant differences (p < 0.05) (analyzed by post-hoc Fisher LSD).

Percent distribution of ARA, EPA and DHA monohydroxides in adipose tissue. Results are expressed as means and standard deviation(SD). Values with different superscript letters in the same row indicate significant difference at p < 0.05 between dietary groups (n=9 per group).

This feature has a sound significance since ARA derived hydroxides are considered more active pro-inflammatory molecules than EPA- and DHA-hydroxides. Many EPA and DHA derivatives have been suggested as signaling molecules and less harmful compounds than the corresponding ω-6 metabolites (47). Furthermore, some of the EPA and DHA derived hydroxides are intermediate monohydroxy biosynthetic pathway markers of SPMs as Rvs, PDs and Mars. According to the predominant action of 15LOX upon EPA found in prediabetic rats, the balance between 15HEPE and its corresponding precursor, the hydroperoxide 15HpEPE, was higher in HFHS-fed rats than in STD-fed rats (values 15HEPE/15HpEPE: 0.77 and 0.55, respectively, **Figure 1B**). This fact could indicate both, a major formation of the EPA precursor hydroperoxide in prediabetic rats than in STD-fed rats accompanied by activity of the antioxidant enzyme GPx substrate to detoxify the hydroperoxides produced. GPx reduces hydroperoxides into secondary metabolites like hydroxides and catalyzes the detoxification of harmful oxygen radicals (20).

Significant production of leukotrienes in adipocytes is associated to advanced steps of the insulin resistance process (9). Consequently, leukotrienes as Leukotriene B4 (LTB4) produced from activity of 5LOX upon ARA were not detected in adipose tissue of prediabetic rats (**Table 4**). Activity of 5LOX and 12LOX upon ARA produced the hydroxides 5HETE and 12HETE, together to the oxo-derivatives 5oxoHETE and 12oxoHETE in prediabetic rats. Therefore, LOX activity upon ARA in adipose tissue of prediabetic rats seemed to be preferentially modulated towards the production of 5HETE (via GPx activity) and 5oxoHETE (via DHO activity) rather than the production of leukotrienes (via the consecutive activities of 5LOX, 5LOX–activating protein, and leukotriene A4 hydrolase) during the first steps of incipient diet-induced diabetes.

Consumption of the HFHS diet differentially affected the formation of lipid mediators produced from non-enzymatic free radical peroxidation. The relative amounts of non-enzymatic hydroxides regarding the total hydroxides formed, resulted in a preferential formation of EPA-derivatives over ARA-derivatives in HFHS-fed animals. Particularly, non-enzymatic 11HETE counted 22.7% of total ARA hydroxides for STD-fed rats and remained 21.8% for HFHS-fed rats (**Figure 1C**). However, the formation of 11HEPE derived from EPA counted 12.7% of total EPA hydroxides for STD-fed rats and resulted significantly increased as for 22.3% in HFHS-fed rats, even though the contribution of EPA was lower in HFHS adipose tissue than STD one (**Table 5**). Accordingly, the total contribution of non-enzymatic EPA derived hydroxides (11HEPE and 18HEPE) to the total EPA hydroxides was higher in HFHS-fed rats than STD ones. Both hydroxides counted 39.9% of total EPA derived hydroxides in STD rats against 51.9% for HFHS rats. Interestingly, 11HEPE and 18HEPE are intermediate monohydroxy biosynthetic pathway markers of RvE1. RvE1 has powerful pro-resolving and insulin-sensitizing actions in the vasculature and metabolic organs and its therapeutic potential for immunometabolic alterations associated with type 2 diabetes has been largely suggested (48).

The formation of DHA hydroxides related to the total hydroxides was found also to increase in HFHS-fed rats versus STD ones **(****Table 5****)**. The proportion of DHA versus ARA and EPA slightly increased in the adipose tissue of prediabetic rats according to the higher value for DHA synthesis mediated by desaturase Δ6D = [22: 6 ω-3/22: 5 ω-3]. Additionally, adipose tissue also offered a set of markers of DHA-resolvers of inflammation. As for EPA metabolites, HFHS diets shifted the LOX activity upon DHA to ARA addressing a greater proportional formation of SPMs precursors (**Table 5**). So, the relative formation of 17HDoHE derived from the subsequent activities of 15LOX, GPx and 5LOX upon DHA and which is precursor of PDX and RvDs, was significantly higher in HFHS-fed rats than in STD (**Figure 1D**). Additionally, combined activities of 12LOX and GPx upon DHA stimulated the formation of 14HDoHE, a precursor of MaRs. Interestingly, sEH is involved in several of these pathways. In particular, sEH catalyzes the formation of MaRs from 14HDoHE and the formation of RvE1 from 11HEPE and 18HEPE. And sEH activity was up-regulated in adipose tissue of these HFHS-fed rats as it is mentioned above.

Finally, PDX was detected in the adipose tissue of both STD- and HFHS-fed rats. But the concentration measured in HFHS prediabetic rats was significantly lower. PDX has demonstrated to ameliorate insulin resistance and inflammation in models of palmitate- or high-fat diet-induced insulin resistance (49). Its anti-inflammatory and antidiabetic effects are linked to attenuate inflammation and insulin resistance in adipocytes *via* an AMPK-dependent pathway (50). Previous works have identified PDs in adipose tissue of rodents (51). Protectins notably decline with obesity, indicating adipose SPMs deficiency, which potentially ending in unresolved inflammation. A similar pattern in which protectins and the precursors 17HDoHE and 18-HEPE, but no RvDs or RvE1 has been described in human adipose tissue depots (10). PDX is a stereo and geometric isomer of PD1. In contrast to PD1, PDX is produced from DHA *via* a di-oxygenation mechanism by 15LOX. Therefore, this pattern could be related with the higher 15LOX activity over DHA found in adipose tissue (**Table 4**). Taken together, these results support that HFHS diet resulted in a lower absolute amount of lipid mediators than STD diet. But, as a homeostasis mechanism to control inflammation, enzymatic and non-enzymatic activities of adipose tissue were modulated to enhance the formation of EPA and DHA derived hydroxides over ARA derived hydroxides. Enhancing the enzymatic activity of LOXs upon DHA and EPA versus ARA helped keep a lower inflammatory lipid mediator profile and stimulate the formation of SPMs in rats suffering from diet-induced prediabetes. Since prediabetic adipose tissue has demonstrated an inflammatory status marked by up-regulation of sEH, formation of PGD2 and reduced levels of epoxides, PGE2 and PDX, these actively coordinated modifications favoring the production of EPA and DHA lipid mediators constitute key mechanisms to restore adipose tissue homeostasis and preserve normal adipose tissue function in those very first steps of the diabetes onset.

#### Effect of Fish Oil on Adipose Tissue

The supplementation with ω-3 PUFAs in STD or HFHS diets, resulted in an enrichment of lipid mediators derived from EPA and DHA, together with a decrease of ARA derivatives (**Table 4**). This is in alignment with the increased concentrations of ω-3 PUFAs detected in adipose tissue (**Table 2**), particularly attributed to a higher proportion of EPA and DHA and lower levels of 18:2 ω-6. For both dietary interventions, the pathway-oriented profiling of lipid mediators in adipose tissues illustrated several routes modulated by the incorporation of ω-3 PUFAs.

According to the higher concentration of DHA achieved through the supplementation with ω-3 PUFAs, STD-fed rats presented an increment of DHA-derived epoxides together with a trend to a decline in the concentration of epoxides derived from ARA. Considering that the relative proportion of ARA in the adipose tissue dropped after the supplementation with fish oil (**Table 2**), this trend to increase DHA epoxides versus ARA epoxides draws the attention to a potential modulation of CYP for DHA in presence of fish oil, probably associated with a substrate competition. Recent research showed that EPA and DHA are highly efficient alternative substrates of CYP enzymes suggesting that the CYP pathway is the dominant pathway in metabolizing ω-3 PUFAs *in vivo (*51). In prediabetic rats fed the HFHS diet, fish oil increased the level of DHA derived epoxides as well (**Table 4**). Previous studies suggest that CYP metabolites as DHA-derived epoxides might play an important role in mediating the anti-cancer and anti-angiogenic effects of ω-3 PUFAs (52).

Considering the activity of sEH, supplementation with fish oil in HFHS-fed rats significantly down-regulated the sEH activity for ARA epoxides reducing the formation of pro-inflammatory ARA di-hydroxides. Therefore, the balance (±)14(15)-DiHET/(±)14(15)-EET was very similar in STD-fed rats with and without ω-3 PUFAs supplementation (0.21 in STD vs 0.20 in STD+ω3 rats) but was significantly reduced in HFHS-fed rats (0.31 in HFHS vs 0.00 in HFHS+ω3rats).

As regards DHA epoxides and their corresponding di-hydroxides, ω-3 PUFAs feeding did not significantly influence the conversion of (±)19(20)-EDP to (±)19(20)-DiHDPA in STD-fed rats. However, ω-3 PUFAs provoked an increase of (±)19(20)-EDP in prediabetic rats fed the HFHS diet. Its corresponding di-hydroxide, (±)19(20)-DiHDPA, was not detected. Therefore, in agreement with ARA results, sEH activity for DHA was found down-regulated in the prediabetic rats supplemented with ω-3 PUFAs. Accumulating evidence suggests the combination of ω-3 PUFAs intake and sEH inhibition as potent anti-inflammatory strategies. Recent studies have provided evidence that ω-3 epoxides and sEH inhibition regulate autophagy and endoplasmic reticulum stress in insulin-sensitive tissues, and modulate inflammation in obese adipose tissue and liver (53). sEH inhibitors in combination with a ω-3 rich diet have demonstrated to contribute to lowering systolic blood pressure and attenuating inflammation in angiotensin-II-dependent hypertension as well (54).

The formation of PGD2 and PGE2 was also modulated in both STD- and HFHS-fed rats. ω-3 PUFAs supplementation in STD-fed rats showed a trend to decrease PGE2 while increasing PGD2. As a result, the balance PGD2/PGE2 was found enhanced (**Table 4**). In the HFHS animal model, supplementation with ω-3 PUFAs showed a diminution of both prostaglandins and a tendency towards upper values of the ratio PGD2/PGE2 as well. The effect of fish oil for ameliorating the production of prostaglandins and leukotrienes has been widely suggested (55). *Ex vivo* experiments carried out on peripheral blood mononuclear cells (PBMCs) has shown that dietary intake of a fish oil rich in DHA decreased the release of PGE2 and pro-inflammatory cytokines as well as the myeloid growth factor G-CSF (55). However, COX-2 may have pro-inflammatory and anti-inflammatory properties depending on the different expression of downstream PGH2 isomerases. A shift from PGD2 to PGE2 formation, and hence a decrease in PGD2/PGE2 balance, has been associated with the progression of inflammatory disorders (56). The results for the STD group supplemented with ω-3 PUFAs agreed with previous studies in which PGD2 has been found overexpressed in asymptomatic inflammatory situations, associated with NF-kappaB inactivation and MMP-9 suppression, whereas PGE2 pathway was significantly prevalent in symptomatic inflammatory conditions (56). Additionally, Fergusson et al. (57) have recently suggested that ω-3 PUFAs supplementation prior to inflammatory stress may lead to dis-inhibition of prostaglandin genes that are normally down-regulated during inflammation. Therefore, ω-3 PUFAs supplementation has demonstrated to up-regulate prostaglandin synthase gene PTGDS, which expresses the rate-limiting enzyme in the production of PGD2 and is required for integrated inflammatory responses to stress induced by endotoxemia in human adipose tissue (57).

Enrichment of EPA and DHA through ω-3 PUFAs supplementation provoked a general decrease in the amount of ARA hydroxides/mg of adipose tissue derived from the consecutive action of LOX and GPx. This decrement affected all the enzymatic ARA hydroxides in STD-fed rats, including 12HETE and 20HETE derived from CYP. And it significantly affected 12HETE and 20HETE in HFHS-fed rats. Meanwhile, the proportion of hydroxides derived from EPA and DHA increased (**Table 5**).

The amount of EPA hydroxides/mg of adipose tissue increased after ω-3 PUFAs supplementation. The only exception was 5HETE in HFHS-fed rats. Concerning DHA hydroxides, supplementation with ω-3 PUFAs of both STD and HFHS groups resulted in a LOX-derived hydroxide pattern similar to the corresponding controls, being 17HDoHE the main hydroxide formed followed by 14HDoHE. The amount of DHA hydroxides/mg of adipose tissue increased after ω-3 PUFAs consumption in both dietary frameworks. 11HDoHE showed the highest increment in both STD- and HFHS-fed rats compared to their corresponding controls.

Particularly, a detailed analysis of hydroxides derived from the action of 5LOX/GPx, 12LOX/GPx and 15LOX/GPx on ARA, EPA and DHA showed that ω-3 PUFAs consumption in STD-fed rats resulted in the following features:

a) Lesser formation of 15HETE than 5HETE and 12HETE.
b) Higher formation of 5HEPE and 12HEPE than 15HEPE.
c) Higher formation of 11HDoHe than other DHA-derived hydroxides.

Therefore, in a STD-diet framework, ω-3 PUFAs might down-regulate the activity of 15LOX upon ARA much more than 12LOX and 5LOX. And ω-3 PUFAs might stimulate the activity of 5LOX and 12LOX upon EPA over 15LOX. As for DHA, ω-3 PUFAs seemed to enhance the activity of 12LOX over the other LOXs. Consequently, in adipose tissue of healthy rats, results addressed a down-regulation of 15LOX and up-regulation of 5 and 12LOX mediated by ω-3 PUFAs.

Supplementation with fish oil produced a higher balance between 12HEPE and its corresponding precursor, the hydroperoxide 12HpEPE, and a higher balance between 15HEPE and its corresponding precursor, the hydroperoxide 15HpEPE as compared to the STD control (**Figure 1B**). These findings pointed out the GPx activity in HFHS-fed rats supplemented with ω-3 PUFAs to detoxify hydroperoxides. The inclusion of EPA and DHA into the STD and HFHS feeding of female Wistar rats has triggered significantly higher activities of plasma antioxidant enzymes including GPx (9).

In HFHS-fed rats, ω-3 PUFAs supplementation contributed to increase the previously observed shift towards EPA-derived hydroxides over ARA hydroxides (**Figure 1A**). The supplementation with fish oil provoked a higher balance between 12HEPE/12HETE and 15HEPE/15HETE in HFHS+ω3 PUFAs group than in STD+ω3 PUFAs group. Therefore, the potential compensatory mechanism aimed to favor the production of less inflammatory EPA metabolites over ARA-derived compounds in prediabetic rats appeared to be significantly strengthened with supplementation of fish oils in a balanced proportion EPA/DHA.

The detailed analysis of hydroxides derived from the action of 5LOX/GPx, 12LOX/GPx and 15LOX/GPx on ARA, EPA and DHA showed that ω-3 PUFAs consumption in HFHS-fed rats resulted in the following features:

a) Lesser formation of 12HETE than 5HETE and 15HETE.
b) Higher formation of 12HEPE than 15HEPE. 5HEPE was not detected.
c) Higher formation of 11HDoHe than 4HDoHe, 14HDoHe, and lesser for 17HDoHe.

According to this predominant action of 12LOX upon EPA than 15LOX, the balance between 12HEPE and its corresponding precursor, the hydroperoxide 12HpEPE, increased in greater proportion as compared to HFHS controls than the balance 15HEPE/15HpEPE (the increment of 12HEPE/12HpEPE was 240% but barely 75% for 15HEPE/15HpEPE) (**Figure 1B**). Therefore, in prediabetic rats, regular consumption of ω-3 PUFAs shifted the activity of LOXs to EPA versus ARA, enhancing the activity of 12LOX over the other LOXs. Additionally, ω-3 PUFAs enhanced the activity of 5LOX and 12LOX upon DHA over 15LOX as well. Collectively the data reveals that ω-3 PUFAs supplementation of the diet-induced prediabetes is marked by promotion of 12LOX activity upon EPA and DHA and further amplification of the anti-inflammatory cascade. Such preference could be associated with substrate competition. Growing evidence suggests a role for 12LOX and 15LOX activities in obese adipose tissue driving chronic local inflammation and metabolic dysfunction (58). Disruption of normal 12LOX and 15LOX functions was associated with adipocyte dysfunction, insulin resistance and diabetes. Emerging research points towards a significant requirement for 12LOX activity in adipocytes for adipocyte differentiation.

Formation of non-enzymatic hydroxides derived from ARA (11HETE) was inhibited after ω-3 PUFAs supplementation in STD-fed rats (**Table 4**). And formation of non-enzymatic hydroxides derived from EPA (11HEPE and 18HEPE) was favored. Interestingly, the formation of 11HETE was not significantly affected after ω-3 PUFAs supplementation in HFHS-fed rats, but fish oil increased the formation of EPA non-enzymatic hydroxides in these prediabetic rats. Therefore, the intermediate monohydroxy biosynthetic pathway markers of RvE1, 11HEPE and 18HEPE were found increased in both, STD- and HFHS-fed rats supplemented with ω-3 PUFAs. 17HDoHE, as a precursor of PDX and RvDs, and 14HDoHE were found significantly enhanced in both diet groups as well. Noteworthy, the amount of PDX was found significantly augmented in all animals supplemented with ω-3 PUFAs.

This shift towards a lower inflammatory state described by fatty acid profiles and lipid mediators’ synthesis in ω-3 PUFAs supplemented groups compared with controls was highly in agreement with the lower values of plasma IL-6, leptin and balance AST/ALT reported in the liver (**Table 1**) (20, 24). Additionally, this anti-inflammatory condition attributed to supplementation with fish oils was associated with decreased lipid deposition in plasma and organs, and the lower plasma insulin values for 67% of the rats fed the HFHS diet.

## Conclusions

Diet-induced prediabetes resulted in general ectopic lipid deposition in plasma, tissues and organs of Sprague-Dawley rats fed HFHS diets. These results were in agreement with the worsen of insulin sensitivity and the increment of perigonadal white adipose tissue detected in these rats after 21 feeding weeks. Adipose tissue of prediabetic rats accumulated lipids with enrichment in palmitic and oleic acids, both of them associated with the risk of developing insulin resistance. Accordingly, consumption of the hyperenergetic diet resulted in an increment of plasma and adipose tissue stearoyl-CoA desaturase 1 indexes, favoring the storage of fat. Meanwhile, prediabetic adipose tissue of HFHS-fed rats showed a pro-inflammatory state associated with an up-regulation of sEH, a trend to release PGD2 together to reduced levels of PGE2 and PDX. In an attempt to control the inflammatory response initiated, LOX and non-enzymatic activities appeared stimulated for EPA and DHA versus ARA. Enhancing the enzymatic activity of LOXs upon DHA and EPA resulted in a lower inflammatory lipid mediator profiling and the formation of intermediate hydroxides precursors of SPMs. This compensatory mechanism to achieve restoration of tissue homeostasis was significantly strengthened through supplementation with fish oils. Thus, increasing proportions of ω-3 PUFAs in adipose tissue significantly stimulated the formation of DHA epoxides by cytochrome P450, enhanced the activity of 12LOX upon EPA and DHA, promoted a higher release of non-enzymatic EPA metabolites and favored the formation of SPMs. Additionally, ω-3 PUFAs supplementation led to a lower ω-6/ω-3 index and reduced sEH and SCD-1 activities in adipose tissue avoiding the accumulation of pro-inflammatory lipids. As a result of this anti-inflammatory condition, fish oils decreased ectopic lipid deposition and contributed to lower plasma insulin values for 67% of the rats fed the HFHS diet. Taken together, these actively coordinated modifications appeared as key mechanisms to restore adipose tissue normal function.

In conclusion, our data suggest a clear compensatory mechanism in prediabetic adipose tissue aimed to restore the anti-inflammatory state through a specific modulation of the production of lipid mediators. Data supported that this homeostasis mechanism is reinforced through the supplementation of the diet with fish oils having high and balanced contents of EPA and DHA. The study highlights new insides on the targets for effective treatment of incipient diet-induced diabetes and the mechanism underlying the potential anti-inflammatory action of marine lipids. These findings can have a strong impact for the development of nutritional strategies and for the right design of nutritional supplements based on fish oils.

## Data Availability Statement

The original contributions presented in the study are included in the article/**Supplementary Material**. Further inquiries can be directed to the corresponding author.

## Ethics Statement

The pertinent permission for this specific study was obtained from the CSIC (Spanish Research Council) Subcommittee of Bioethical Issues and the regional Catalan authorities (reference number DAAM7921).

## Author Contributions

Conceptualization, GD, JT, MN and IM. Data curation, GD and SL. Formal analysis, GD, LM and IM. Funding, IM. Methodology, GD, SL and IM. Supervision, IM. Validation, GD, SL and IM. Writing – original draft, IM. Writing – review and editing, GD, SL, LM, NT, MN, SR-R, JT and IM. All authors contributed to the article and approved the submitted version.

## Funding

This work was supported by the Spanish Ministry of Science and Innovation (grants AGL2013-49079-C2-1-R and RTI2018-095659-B-I00).

## Conflict of Interest

The authors declare that the research was conducted in the absence of any commercial or financial relationships that could be construed as a potential conflict of interest.

The reviewer AG-I declared a shared affiliation with several of the authors (IM, GD, LM, JT and SR) to the handling editor at the time of the review.
