# Supplementary material for: Fish Oil Improves Pathway-Oriented Profiling of Lipid Mediators for Maintaining Metabolic Homeostasis in Adipose Tissue of Prediabetic Rats
Source: Front Immunol. 2021 Apr 21;12:608875. doi: 10.3389/fimmu.2021.608875 (PMC8097180; doi:10.3389/fimmu.2021.608875)
Supplement: Supplementary file 1 [file DataSheet_1.docx]

Table S1: Diet composition. ST (standard diet), HFHS (high-fat high-sucrose diet), ω3 (supplementation with fish oil).

|  | STD | STD + ω3 | HFHS | HFHS + ω3 |
| --- | --- | --- | --- | --- |
| Flour (g) | 1000^a^ | 1000^a^ | 1000^b^ | 1000^b^ |
| Porcine gelatin (g) | 25 | 25 | 25 | 25 |
| Soybean lecithin (g) | 6 | 6 | 22 | 22 |
| Oil (mL) | 19 mL soybean | 19 mL EPA:DHA 1:1 | 24 mL soybean | 24 mL EPA:DHA 1:1 |
| Protein (% weight) | 16.4 | 16.4 | 21.7 | 21.7 |
| Fat (% weight) | 6.2 | 6.2 | 24.1 | 24.1 |
| Carbohydrates (% weight) | 46.6 | 46.6 | 45.0 | 45.0 |
| Total energy density (kcal/g) | 3.1 | 3.1 | 4.8 | 4.8 |
|  |  |  |  |  |

^a^Teklad Global 14% Protein Rodent Maintenance Diet (Envigo, IN, USA), ^b^TD.08811 45% Kcal Fat Diet (Envigo, IN, USA).

Table S2: Composition of the fatty acid diet. ST (standard diet), HFHS (high-fat high-sucrose diet), ω3 (supplementation with fish oil). Results are expressed as a percentage of total fatty acids (mg/100mg of Total FA).

| FATTY ACIDS | STD | STD + ω3 | HFHS | HFHS + ω3 |
| --- | --- | --- | --- | --- |
| 14:00 | 0,0 | 0,1 | 11,5 | 11,6 |
| 16:00 | 14,8 | 14,6 | 32,2 | 32,0 |
| 16:1ω7 | 0,0 | 0,1 | 1,7 | 1,7 |
| 18:00 | 2,9 | 2,9 | 13,7 | 13,7 |
| 18:1ω9 | 20,5 | 20,3 | 26,4 | 26,2 |
| 18:1ω7 | 0,0 | 0,0 | 0,7 | 0,7 |
| 18:2ω6 | 58,6 | 57,8 | 9,7 | 8,7 |
| 20:00 | n.d. | 0,0 | n.d. | 0,0 |
| 18:3ω3 | 2,9 | 2,9 | 1,1 | 1,1 |
| 20:1ω9 | 0,0 | 0,0 | 0,0 | 0,0 |
| 18:4ω3 | 0,0 | 0,0 | 0,0 | 0,0 |
| 20:2ω6 | 0,0 | 0,0 | 0,0 | 0,0 |
| 20:3ω6 | n.d. | 0,0 | n.d. | 0,0 |
| 20:4ω6 | 0,0 | 0,0 | 0,0 | 0,0 |
| 22:1ω11 | 0,0 | 0,0 | 0,0 | 0,0 |
| 22:1ω9 | 0,0 | 0,0 | 0,0 | 0,0 |
| 20:4ω3 | 0,0 | 0,0 | 0,0 | 0,0 |
| 20:5ω3 | 0,0 | 0,4 | 0,0 | 0,5 |
| 24:1ω9 | 0,0 | 0,0 | 0,0 | 0,0 |
| 22:5ω3 | 0,0 | 0,1 | 0,0 | 0,1 |
| 22:6ω3 | 0,0 | 0,4 | 0,0 | 0,6 |
| Total SFAs | 17,5 | 18,3 | 59,4 | 60,5 |
| Total MUFAs | 21,1 | 20,3 | 29,3 | 28,4 |
| Total PUFAs | 61,1 | 61,0 | 10,8 | 10,7 |
| Total ω3 | 3,3 | 3,2 | 1,6 | 1,4 |
| Total ω6 | 58,8 | 58,9 | 10,4 | 10,6 |

|  |
| --- |
